# Effect of Dietary Regimen on the Development of Polycystic Ovary Syndrome: A Narrative Review

**DOI:** 10.7759/cureus.47569

**Published:** 2023-10-24

**Authors:** Salma Alomran, Edric D Estrella

**Affiliations:** 1 Preventive Medicine, Ministry of Health, Al-Ahsa, SAU; 2 Public Health, College of Applied Medical Sciences, King Faisal University, Al-Ahsa, SAU

**Keywords:** dietary regimen, diet, prevention, pcos, polycystic ovarian syndrome

## Abstract

Background: Polycystic ovary syndrome (PCOS) is a common endocrine disorder affecting 4%-20% of women worldwide. The pathogenesis of PCOS is still unconfirmed. Some risk factors for the disease are obesity, insulin resistance, genetic factors, and diet.

Aim: Our aim is to review studies investigating the role of diet in the development of PCOS.

Method: We looked into studies published in different databases, such as PubMed, Scopus, Google Scholar, and Web of Science, using specific keywords as per our study topic.

Results: High-carbohydrate, high-fat diets, low-fiber diets, high glycemic index and glycemic load, and Western diets were associated with a higher risk of PCOS. Some vitamins, such as Vitamin D and B9, the Dietary Approaches to Stop Hypertension (DASH) diet, fruits, nuts, and seeds, such as pumpkin and sunflower, are associated with a lower risk of PCOS. The Alternate Healthy Eating Index-2010 (AHEI-2010) diet reduces the risk of hyperandrogenic or oligoanovulatory phenotypes. The review revealed that unhealthy diets encompass high levels of carbohydrates, animal proteins, fats, and processed foods. Despite inconsistent results from certain studies claiming no disparity in the dietary patterns between PCOS patients and healthy controls, the majority of researchers have amassed sufficient evidence linking nutrition to the incidence of PCOS. The review also underscores the significance of the interplay between genes and the environment in the prevalence of PCOS. Individuals possess a genetic predisposition to the condition from birth, and subsequent exposure to detrimental environmental factors, particularly diet and inactivity, trigger epigenetic changes that contribute to the development of the disorder. This study further illuminated the existence of the "lean PCOS" phenomenon, wherein roughly 20% of global PCOS cases exhibit clinical manifestations of the syndrome but maintain a normal or below-average weight.

Conclusion: To sum up, the collective body of assessed research indicates that women with PCOS tend to share similar dietary habits, characterized by the consumption of numerous unhealthy foods such as processed foods, animal proteins, carbohydrates, and fats. While some studies present conflicting findings, these contradictions underscore the necessity for further investigation employing extensive cohorts.

## Introduction and background

Polycystic ovary syndrome (PCOS) is a widespread endocrine disorder affecting 4% to 20% of reproductive-age women worldwide [1]. It is characterized by varying signs and symptoms, including amenorrhea, infertility, hirsutism, and obesity [2]. The condition is also linked to a higher risk of insulin resistance and diabetes, a higher cardiovascular risk profile, and mental health problems [3, 4]. Rotterdam Criteria were set to facilitate the diagnosis of PCOS. The diagnosis according to this criteria requires the presence of two out of three following conditions: (1) oligomenorrhea or anovulation; (2) clinical and biochemical hyperandrogenism; and (3) polycystic ovaries (12 or more follicles in each ovary measuring 2-9 mm) [5].

The prevalence of PCOS varies by region, highlighting the global burden of the disease. According to the Lancet Regional Health, Europe exhibits a rate of 276.4 cases of PCOS per 100,000 women [6]. The CDC states a 6%-12% prevalence in the U.S., equivalent to up to five million women [7]. In a study conducted in 2017, the age-standardized incidence was highest in Andean Latin America at 220.50 per 100,000, followed by high-income areas of Asia Pacific at 151.10 per 100,000, and the Caribbean at 140.15 per 100,000 [8]. Conversely, the incidence was lowest in southern Latin America, at 52.91 per 100,000 people. Tropical Latin America demonstrated the most significant increase between 2007 and 2017, with a rise of 4.29%. Similarly, East Asia experienced a rise in the age-standardized incidence of 3.70%, and eastern Sub-Saharan Africa at 2.76% [8]. In contrast, a decline in age-standardized incidence was noted between 2007 and 2017 in North Africa and the Middle East, decreasing by 0.61%, as well as in Southern Latin America, declining by 0.22% [8]. The rate of disability-adjusted life years (DALYs) was highest in Andean Latin America at 57.66 per 100,000, followed by the Caribbean at 38.53 per 100,000 and the high-income Asia Pacific at 36.57 per 100,000 women, respectively [8]. These statistics underscore the severity of the global burden of PCOS, emphasizing the necessity to comprehend its etiology and pathogenesis and, consequently, to develop effective preventive and management protocols.

The exact pathogenesis of PCOS remains unclear; it is likely caused by multiple factors, including environmental and genetic ones [1]. One theory posits that excess androgen production in early life leads to epigenetic changes that can interact with environmental factors, such as diet and obesity, resulting in the development of PCOS [9]. The first-line treatment for PCOS is lifestyle modification in the form of healthy eating habits and physical activity to achieve weight loss [10]. According to the Nurse Health Study Cohort, adherence to a fertility diet reduces the attributable risk of infertility related to ovulatory disorder by 66% (95% CI: 29%-86%) [11]. Advanced glycation end products (AGEs) and oxidation proteins, products of cooking at high temperatures, and unhealthy diets were found to be higher in women with PCOS than in healthy women [12, 13]. The actual relationship between an unhealthy diet and the development of PCOS is still unconfirmed, and the findings are contradictory.

Understanding the risk factors for PCOS is essential to aid in the prevention and control of this disease. The influence of diet on the development of PCOS is still debated. To our knowledge, there are several studies that have investigated the association between dietary patterns and food groups and the development of PCOS. Hence, our aim in this narrative review is to examine and discuss the evidence regarding the effect of dietary patterns on the development of PCOS. The overarching research question is, ‘What is the effect of dietary patterns on the development and prevalence of polycystic ovary syndrome (PCOS)?’

## Review

Methodology

Studies were identified by searching the electronic databases PubMed, Scopus, Google Scholar, and Web of Science for studies published after 2010 and before 2022. These databases were selected because they provide scholarly, peer-reviewed journal articles with credible findings for evidence-based practice (EBP). The publication period between 2010 and 2022 was chosen to provide sufficient scholarly data, especially demonstrating scientific research over a long period to note any inconsistencies in findings and acknowledge the development of new perspectives over time. A search was performed by using a combination of keywords relevant to PCOS, diet, and lifestyle. Studies from various countries were included in the review based on population, intervention, comparison, and outcome. Studies selected for review included original research articles, systematic reviews, and meta-analyses in which 1) the primary objective was to compare the type of diet between women with and without PCOS, and 2) enrollment exceeded 10 participants in each study arm. The results are presented in Table 1, and a discussion is made in the subsequent section.

**Table 1:** Summary of studies PCOS: polycystic ovary syndrome; MEDLINE: Medical Literature Analysis and Retrieval System Online; Embase: Excerpta Medica Database; CINAHL: Cumulative Index to Nursing & Allied Health; DALYs:  disability-adjusted life years

Author (Year)	Study Design	Sample, Sample Size	Methodology	Findings
Deswal et al. (2020)	A brief systematic review	The study included 27 surveys, with 32,125 participants in total.	Computer-assisted search on the leading databases, including PubMed, MEDLINE, Embase, Wiley, Google Scholar, ScienceDirect, and ISI Web of Knowledge. Keywords used included “prevalence of PCOS,” “PCOS in reproductive age,” “Polycystic Ovary Syndrome,” and “Epidemiology of PCOS,”	The review revealed that one in 10 women (10%) has PCOS globally. Overall, girls with PCOS presented with higher instances of oligomenorrhea, and infertility was more common among women with PCOS. The authors found that PCOS affects both obese and lean women, but the prevalence was higher among obese cases with insulin resistance.
Sedighi et al. (2015)	Descriptive comparative study	Sixty-five women with PCOS, with a control of 65 women without the disease. Ages ranged from 18 to 45 years. The selection was done through multi-stage randomization.	Data collection entailed questionnaires to examine diet, physical activity, and unhealthy behaviors. Physical activity was gauged using the International Physical Activity Questionnaire.	A significant association was found between an unhealthy diet and a lack of physical activity and the incidence of PCOS (p=0.009). However, the authors did not find a relationship between the disease and other types of unhealthy habits.
Altieri et al. (2013)	Case-control study	A hundred women with PCOS, either obese or overweight, and 100 controls matched for age and BMI	Dietary habits were examined using the seven-day food diary, and fasting hormones and other metabolic indicators were tested for all the participants.	No major difference in the diet was observed between the test and control groups in terms of macronutrients, energy, and glycosylated end products. However, women with PCOS were found to be consuming less energy from lipids and exhibited a higher intake of fiber. The test group also demonstrated a higher consumption of cheese, high-glycemic index sweets, and raw oil instead of cooked fats when compared to the controls. There was also a considerable difference between the test and control in regard to the relationship between dietary habits and metabolic parameters, especially high-density lipoprotein (HDL)-cholesterol and insulin.
Xenou & Gourounti (2021)	Systematic review	Seven out of the 123 retrieved articles proved relevant to the study objectives.	A systematic search of research articles in electronic databases using medical topics was done.	The findings indicated that diet has a crucial role in the clinical manifestation and laboratory findings of PCOS. A change in diet was associated with an improvement in the clinical aspect of the condition.
Mani et al. (2013)	Retrospective cohort study	The study included 2,301 women with PCOS with a mean age of 29.6.	The participants were followed for 20 years, with a total of over 12,000 person-years by the end of the follow-up. The main focus was on type II diabetes mellitus (T2DM), myocardial infarction (MI), heart failure, angina, stroke, and cardiovascular-related death.	There was a 3.6 incidence of T2DM per 1000 person-years. The incidence of Mi per 1000 person-years was 0.8 for angina; it was 1.0 for heart failure (HF), 0.3 for stroke, 0.0, and 0.4 for cardiovascular (CV)-related death. The prevalence of MI and angina increased with age, with those above 65 years having a 27.7% rate of both conditions. In addition to age, a history of hypertension and smoking were significantly correlated with CV events in women with PCOS.
Cooney et al. (2017)	A comprehensive systematic review and meta-analysis	Thirty cross-sectional studies were included in the systematic review, with a total of 3,050 women with PCOS and 3,858 healthy controls drawn from 10 countries. 18 studies were included in the meta-analysis (MA) of depressive symptoms, and nine studies were included in the MA on anxiety symptoms.	A thorough search was done on databases, including Cochrane, Ovid, PsychInfo, and Embase. An estimation of the pooled odds ratio of anxiety and depressive symptoms was done using random effects MA.	The findings indicated increased odds of depressive and anxiety symptoms among women with PCOS even after matching the participants for BMI. The studies lacked substantial heterogeneity in the MA regarding overall depressive symptoms, but heterogeneity was observed for anxiety symptoms. Women with PCOS who also had depression were found to have higher mean values of hirsutism, BMI, and insulin resistance, while those with concurrent anxiety were found to have higher mean values of hirsutism, BMI, and free testosterone.
Lin & Lujan (2014)	A review of the literature	Ten studies published between 1990 and 2014 were included in the review.	A literature search in electronic databases was done. The sites included CINAHL, PubMed, and PsychInfo, using keyword combinations related to PCOS and diet, nutrition assessment, and lifestyle.	The findings did not indicate a significant relationship between dietary habits and the incidence of PCOS when data was collected without adjusting for body composition. The seven-day food analysis revealed a high intake of energy foods among women with PCOS compared with controls. There was a positive correlation between carbohydrate consumption and FSH levels. There were mixed findings regarding the impact of physical activity, with some studies showing no difference between test and control groups, while others demonstrate some degree of positive association.
Cutillas-Tolin et al. (2021)	Case-control study	The study included 126 PCOS cases and 159 controls. The PCOS diagnosis entailed hyperandrogenism, polycystic ovary morphology, and oligo anovulation	Valid food frequency questionnaires were used for data collection regarding five dietary indicators: Alternate Healthy Eating Index (AHEI), AHEI-2010, alternate Mediterranean Dietary Score (aMED), relative Mediterranean Dietary Score (rMED), and Dietary Approaches to Stop Hypertension (DASH).	The AHEI-2010 index exhibited an inverse association with hyperandrogenism and oligoanovulation. No statistically significant association was established between dietary indices and total anovulatory or ovulatory PCOS.
Eslamian et al. (2017)	Case-control study	The study had 281 women with PCOS and 472 controls matched for age.	Interviews were conducted between February 2012 and March 2014 at clinics in Tehran, Iran, using the validated semi-quantitative food frequency questionnaire. The scores of interest were glycemic load (GL) and average dietary glycemic index (GI). Total intake of carbohydrates, refined grains, fiber, and whole grains was also assessed.	The mean dietary GI values were 59.7 with 5.9 SD for the test group and 51.8 with 4.7 SD for the control group. The GL values were 173.6 (39.1 SD) for the test and 155.34 (35.2 SD) for the controls. The consumption of fiber was inversely related to PCOS. The findings indicated that high dietary GI and GL with a low intake of fiber have a significant association with PCOS.
Eslamian & Hekmatdoost (2019)	Case-control study	The study included 281 women with PCOS and 472 controls	Interviews were conducted between February 2013 and March 2015 in endocrine clinics in Tehran, Iran, using validated semi-quantitative food frequency questionnaires to investigate dietary intakes.	The analysis yielded two nutrient patterns: High loads of micronutrients, including niacin, riboflavin, thiamin, pyridoxine, pantothenic acid, magnesium, cobalamin, folate, vitamin D, vitamin C, total fiber, vitamin E, selenium, phosphorus, mono and polyunsaturated fatty acids, manganese, potassium, vitamin K, and vegetable proteins. High loads of macronutrients, including animal protein, carbohydrate, cholesterol, and fat, plus other micronutrients like sodium, iron, biotin, copper, fluoride, calcium, and zinc. Participants in the highest tertile of the second category had a higher risk of PCOS, while those in the highest tertile of the first group had the lowest risk, thus indicating significant interactions between dietary intakes and PCOS.
Lin et al. (2019)	Case-control cross-sectional analysis	Eighty women with PCOS and 44 controls	Dietary intake and quality were assessed using the Healthy Eating Index 2015, while physical activity was evaluated using questionnaires and waist-worn accelerometers.	Women in the test group satisfied the dietary references for proteins, carbohydrates, and fat but were below the recommendations for vitamin D, vitamin B9, total fiber, and sodium. They met the US guidelines for physical activity. No significant differences were observed between the test and control groups regarding physical activity and dietary behaviors. However, failure to have unique targets for dietary and PA interventions was considered to support the recommendations for a healthy lifestyle in managing PCOS.
Shahdadian et al. (2019)	Case-control	The study included 225 newly diagnosed PCOS patients and 345 healthy controls in Isfahan, Iran	Assessment of dietary intake was done using a 168-item food frequency questionnaire. Principal component analysis was used to identify dietary patterns.	There were three dietary patterns: Western foods, including animal proteins, carbohydrates, fat, and processed foods, among others; Plant-based: including vegetables, fruits, legumes, nuts, fruit juices, and condiments, among others; Mixed diet – including components from both Western and plant-based categories. The higher tertile of the Western category was associated with the highest risk of PCOS, while the higher tertile of plant-based foods had increased odds compared to the lowest tertile. There was a 66% lower likelihood of developing PCOS for people in the second tertile of the mixed dietary category when compared to the lowest one. The findings indicated a high risk of PCOS for Western and plant-based dietary patterns, while moderate adherence to the mixed category demonstrated a lower risk of the condition.
Shishehgar et al. (2016)	Case-control study	The study was on 142 women with PCOS and 140 healthy controls. Set in Shahid Beheshti University of Medical Sciences, Tehran, Iran.	A comparison of dietary intakes was done using a food frequency questionnaire (FFQ), and the means between the test and control groups were done using the T-test and Mann-Whitney.	There were similar energy and macronutrient intakes for both the test and control groups, but the PCOS women had a higher intake of high-glycemic index foods and lower consumption of legumes and vegetables as compared to controls.
Hajivandi et al. (2020)	Qualitative study	Thirty-three adolescent women with PCOS from Shiraz, Iran	In-depth interviews, focus group discussions, and field notes were used. The data collected entailed what the girls eat and their food habits, including whether they skip meals and how much they eat in a single serving.	Three categories of food habits emerged: High intake of fatty and salty foods; low consumption of dairy products, fiber, meat, fish, beans, and seafood; and consumption of large meals; poor dietary and physical activity habits; and skipping of meals.
Liu et al. (2021)	Data across 194 countries	Systematic analysis of data showing PCOS incidence and DALYs for 194 countries	The Global Burden of Disease, Injuries, and Risk Factors Study (GDB) for 2017 was analyzed to examine data on the total and age-standardized incidence of disease and DALYs for women within the productive age range over 10 years, from 2007 to 2017.	The incidence was found to be 1.55 million cases of PCOS across the globe, with 0.43 million DALYs. An increase of 1.45% in age-standardized incidence over the 10 years was observed; the rate rose to 82.44 cases per 100,000 population. Similarly, age-standardized DALYs increased by 1.91% over the period; figures rose to 21.96 per 100,000 people.
Kshetrimayum et al. (2019)	A review of the literature	N/A	Scholarly sites were searched to provide relevant data on the role of environmental, occupational, and lifestyle factors affecting PCOS prevalence. The locations included PubMed, Google Scholar, Toxline, and Medline.	Reviewed literature revealed that PCOS has an in-utero origin, and its development later in life is influenced by both endogenous (host) and exogenous factors. The latter include lifestyle, occupation, dietary habits, and environmental factors.
Parker et al. (2022)	A narrative review	N/A	A literature search using relevant terminologies related to the evolutionary, genetic, molecular, and epigenetic basis of PCOS was done.	The results demonstrated significant evidence for an evolutionary basis for PCOS pathogenesis. The findings showed that genetics, epigenetics, molecular biology, and metabolism influence the development and progress of PCOS.
Toosy et al. (2018)	Review of literature	N/A	A literature search from credible sites, including PubMed, Google Scholar, and EBSCOhost using such relevant terms as ‘lean polycystic ovary syndrome,’ OR ‘lean PCOD,’ OR ‘lean polycystic ovarian disease,’ OR ‘hyperandrogenism,’ AND ‘low BMI,’ OR ‘low body mass index.’	Results demonstrated that lean PCOS entails alterations in the hormonal, hematological, and metabolic profiles of affected women. However, the study showed that the derangements were either comparable to those of obese PCOS women or were less severe.

Review findings

In a 2014 systematic review, Lin and Lujan investigated the comparison of dietary intake and physical activity between women with and without PCOS, and they found that the overall energy and nutrient intake were similar between the two groups [14]. These findings were quite similar to the results of Deswal et al., who found that PCOS affects both obese and lean women alike, although the prevalence was higher among obese cases with insulin resistance [1]. However, regarding body mass index (BMI), notable differences in dietary intake were evident, for example, with high glycemic index (GI) foods among women with PCOS. Moreover, many of the reviewed studies have found that women with PCOS consume more energy-rich foods with high fat and low fiber content [14]. However, there was no study incorporating different PCOS phenotypes or age stages into the analysis [14]. The conclusion was that since only a limited number of studies were available in the US investigating the lifestyle of women with PCOS and the findings did not demonstrate a major difference between participants with PCOS and those without, the effects of nutrition on metabolic outcomes should be studied more thoroughly among the PCOS population.

In 2022, Scarfò et al. demonstrated the role of epigenetics in the pathogenesis of PCOS, noting that various factors such as lifestyle, age, environment, and disease state may alter the clinical manifestation in a person due to epigenetic modifications [15]. Particularly, the authors focused on the epigenetic and molecular mechanisms and how their interplay with diet and physical activity influences the presentation of PCOS. Scarfò et al. argue that the role of genetics in PCOS is indisputable, with several genes being involved in the pathophysiology of the disease [15]. They note that, due to gene polymorphism, dissecting the genomic nature of PCOS etiology is significantly complicated. However, researchers have still been able to categorize different gene types into subgroups according to their influence on the secretion and functioning of various hormones. Given the comorbidities involved in PCOS, investigating the genes associated with certain conditions to which patients are vulnerable is crucial. For instance, Calpain-10 (CAPN10), implicated in insulin resistance, has been investigated due to PCOS women's predisposition to glucose intolerance and type 2 diabetes mellitus (T2DM) [15]. Similarly, the insulin receptor (INSR) gene is of interest since it encodes the tyrosine kinase family, a critical group of receptors whose abnormalities induce insulin resistance. Moreover, the abnormal glycemic patterns in PCOS women, closely associated with their predisposition to obesity, are worsened by the INSR rs1799817 polymorphism [15].

Scarfò et al. discovered that epigenetic mechanisms are involved in PCOS pathogenesis, with ample data demonstrating the role of DNA methylation and microRNAs in the onset and progression of PCOS [15]. Particularly, the authors noted that epigenetics could regulate crucial environmental factors, such as diet, and the related incidence of obesity among PCOS women. Additionally, epigenetic regulation influences the development of an adverse intrauterine environment. Regarding DNA methylation, the researchers noted that alterations have been found in affected tissues like the ovaries, skeletal muscle, and adipose tissue in the majority of women with PCOS. Altered DNA methylation has also been detected in blood from the umbilical cord, suggesting a possible association between the PCOS phenotype and epigenetic changes at the cellular level involving both systemic and fetal circulation [15].

Similar findings are demonstrated by Kshetrimayum et al., who observed an interplay between genetic and environmental factors, particularly obesity [15]. The authors argue that without this interaction, the clinical manifestation of PCOS may not occur, even for women who are genetically predisposed to the condition. This finding underscores the role of diet in the pathogenesis of PCOS, as certain foods increase the chances of a woman becoming obese, and if genetics predispose them to PCOS, then the risk of developing the disease is heightened. The authors note that the origin of PCOS can be traced to early life during embryogenesis, and it continues throughout a person’s life cycle, with environmental triggers and lifestyle factors influencing its manifestation in vulnerable women [16]. Among the most significant predisposing factors is diet, which interacts with genetics to increase vulnerability to the disease. Kshetrimayum et al. consider that despite the genetic basis of PCOS not being fully understood, evidence shows that a majority of PCOS women have a family history of diabetes, while some have a personal history of the condition [16]. Since evidence indicates a significant role of genetics in PCOS development but specific causation isn't established due to existing polymorphism and heterogeneity, innovative techniques, including next-generation sequencing, need to be employed in research to enhance understanding of the underlying variants of the condition.

Scarfò et al. discuss the molecular mechanisms of PCOS, noting the elevation of insulin levels in the blood, resulting in the overexpression of mir-93 and inhibition of mir-145. This situation favors cell growth and limits apoptosis [15]. Consequently, there is an increase in proliferation and a decrease in cell death, leading to impaired folliculogenesis and ovulation. Additionally, elevated insulin and insulin growth factors cause hypertrophy of the inner theca cells, resulting in the overproduction of androgens. This effect is exacerbated by the impact on pituitary luteinizing hormone (LH) pulsatility [15]. With significantly elevated androgens, antral follicle development is impaired, leading to anovulation.

Scarfò et al. note that in women with PCOS, alterations in DNA and miRNA methylation, and in some instances, chromatin status, occur in various locations such as blood, adipose tissue, serum, theca cells, and granulosa cells [15]. These epigenetic changes are triggered by an adverse intrauterine environment for a fetus or by environmental factors like diet and postnatal obesity. Thus, a woman can be genetically predisposed to PCOS from birth, and upon exposure to relevant factors, especially diet, they develop the disease.

Parker et al. emphasize the interplay between genetics and diet in PCOS development [17]. The authors argue that genetically susceptible individuals manifest the disease after exposure to nutritional and environmental factors, primarily influenced by contemporary lifestyles. They contend that a mismatch exists between evolutionary survival mechanisms at the genetic level and prevailing lifestyle tendencies, influencing various PCOS phenotypes based on abundant research findings [17]. The authors cite extensive research indicating the role of gene variant-environment interactions in PCOS etiology, highlighting that genetics contribute to less than 10% of disease susceptibility [17]. Hence, they underscore the significance of environmental determinants, especially dietary patterns. Common gene loci corresponding to different PCOS phenotypes have been identified through genetic and genome-wide association studies (GWAS) across diverse ethnic populations. This presentation of PCOS as a polygenic trait underscores that its manifestation depends on interactions between genomic variants and environmental triggers [17]. Parker et al. contend that excess androgens in humans can originate from fetal, placental, or maternal sources, while dietary and environmental factors affect the developmental programming of associated gene variants in PCOS women. The authors highlight the importance of postnatal negative exposure to lifestyle and environmental triggers, including poor dietary patterns and endocrine-disrupting chemicals (EDCs), which activate epigenetically programmed pathways leading to clinical PCOS features.

Deswal et al. found that PCOS pathogenesis is influenced by specific genetic factors associated with key biological pathways [1]. The authors noted that these factors affect pathological outcomes like insulin dysregulation and impaired glucose homeostasis, linked to T2DM and obesity comorbidities in PCOS women. Their findings underscore the critical role of lifestyle in PCOS development and progression among genetically and biologically susceptible women [1]. The authors further note that due to PCOS's multifaceted nature resulting from multiple genetic and environmental factors, its consequences manifest not only in the reproductive system but also in the cardiovascular, immune, metabolic, and psychological health of individuals with the disorder. Understanding PCOS has been advanced by GWAS findings, revealing the complexity of its pathophysiology through the identification of crucial genes involved in gonadotropin action, steroidogenesis, insulin secretion and action, hypothalamic-pituitary pathways, adipose tissue abnormalities, lipid metabolism, homeostasis, and chronic inflammation [1]. Some involved genes include the follicle-stimulating hormone receptor (FSHR), the luteinizing hormone receptor (LHR), INSR, thyroid adenoma-associated (THADA), ERB, and high mobility group A2 (HMGA2). Grasping the role of these genetic factors in predisposing women to environmental effects, particularly implicated dietary patterns, is essential for condition prevention.

Research has demonstrated the impact of specific food types and groups on the prevalence of PCOS. Cutillas-Tolin et al. (2021) assessed the association between dietary indices and PCOS phenotypes in 126 cases of PCOS and 159 controls using the food frequency questionnaire (FFQ) [18]. Five dietary patterns were assessed, including the Alternate Healthy Eating Index (AHEI), AHEI-2010, relative Mediterranean Dietary Score (rMED), alternate Mediterranean Dietary Score (aMED), and Dietary Approaches to Stop Hypertension (DASH) [18]. The AHEI-2010 index was inversely associated with hyperandrogenism + oligoanovulation PCOS phenotype (OR Q3 vs. Q1 = 0.1; 95% CI: 0.0; 0.9; P for trend = 0.02 [18]). The authors hypothesized that the observation was mainly driven by vegetable food items in the AHEI-2010 index [18]. Oligo/amenorrheic phenotype risk was associated with adherence to the DASH diet (p-value for trend = 0.05) [18]. Thus, an association between dietary habits and PCOS was established.

Sedighi et al. discovered a significant association between an unhealthy diet, physical inactivity, and the prevalence of PCOS [2]. Particularly, the study highlighted the importance of specific food types. The researchers found that women whose diets include high-calorie and high-fat components exhibit a relatively high incidence of PCOS. This situation impacts hormone production and metabolism, consequently influencing the manifestation of PCOS. In contrast, individuals consuming healthier food types, such as nuts and fruits, experienced a lower incidence of the disease [2]. Furthermore, a considerable proportion of women with PCOS were observed to consume more greasy foods and cookies compared to healthy controls. Consequently, the authors concluded that adopting a change in dietary behavior could lead to improved clinical status for PCOS cases, including enhanced hormonal health and metabolism [2]. They recommended enhancing patient education to maximize the benefits of nutritional modifications in combating PCOS.

The impact of food groups was also demonstrated by Altieri et al., who noted that women with PCOS consumed a higher proportion of high-glycemic index sweets, saturated fat, and cheese compared to healthy controls [10]. Similar outcomes were observed by Xenou and Gourounti, who found that fruits and vegetables, both non-starchy and low-glycemic index categories, were associated with a lower incidence of PCOS [13]. These categories encompass food items like broccoli, asparagus, bean sprouts, mushrooms, cauliflower, blueberries, citrus fruits, and melon. Other beneficial food items for PCOS include small servings of low-fat dairy products, lean red meat, omega-3 fatty acid-rich fish, poultry, fish oils, olive oils, and whole grains [13]. Additionally, certain seeds like pumpkin, sunflower, and almonds were identified as beneficial for PCOS women. Most of these food items fall within the healthy categories of aMED and DASH [13]. Consequently, the authors concluded that incorporating these food items into one's diet, coupled with a reduction in the consumption of unhealthy food categories, could lead to significant improvements in the clinical status of women with PCOS.

Lin and Lujan's research revealed that energy-dense foods are linked to a higher incidence of PCOS [14]. In their review, the authors emphasized the importance of achieving energy balance in PCOS management. They noted that the most effective interventions involve both dietary measures and weight-loss strategies. Concerning food categories, Lin and Lujan found that high carbohydrate consumption impacts FSH, a critical hormone in follicle growth [14]. Similarly, proteins, total energy intake, and cholesterol levels influence the production of androstenedione, a testosterone precursor.

Eslamian et al. studied women aged 20-35 who were diagnosed with PCOS three years prior to the study [19]. A total of 281 women with incident PCOS and 472 age-matched controls were included. The two groups did not exhibit significant differences in regard to the mean starch and total sugar intakes. In contrast, the mean intakes of carbohydrates, refined grains, glycemic index, and glycemic load were significantly higher among PCOS cases (P<0.05), whereas the controls were found to be consuming significantly more dietary fiber and whole grains (P<0.005) [19]. However, this relationship was weaker when a full adjustment model was applied, indicating a more significant role in other metabolic risks, such as obesity. It is also essential to keep in mind that this study was hospital-based, making it prone to Barkson's bias [19]. Such findings further underscore the lack of consensus on the relationship between diet and PCOS, although guidelines still emphasize the importance of lifestyle change in managing the disease.

In a 2019 study, Eslamian and Hekmatdoost studied 281 women with PCOS and 472 controls aged 20-35, assessing usual dietary intake using validated FFQ dividing food patterns into two categories: micronutrients found primarily in vegetable foods and micronutrients in animal foods, and they found that the risk of PCOS was significantly higher in the second pattern (OR: 2.38, 95% CI: 1.69-3.21) [20]. Conversely, the second food pattern was associated with a lower risk of PCOS (OR: 0.48, 95% CI: 0.21-0.82) [20]. In 2013, Altieri et al. found that women with PCOS had a lower intake of fiber and lipid energy, although no differences in macronutrients, energy, or glycosylated end products were observed between the test and control groups [10]. However, Eslamian and Hekmatdoost’s study could also be limited by the risk of Barkson's bias [20].

In a 2019 study, Lin et al. found people with PCOS to be similar to controls in terms of their carbohydrate, protein, and fat intake, but they also found that women in the test group did not meet the recommended dietary reference intakes for vitamin D, vitamin B9, total fiber, or sodium [21]. The authors sought to compare and contrast the dietary and physical activity tendencies of 80 women with PCOS against the control of 44 PCOS-free women. Among the parameters that they focused on were diet, diet quality, and physical activities [21]. Although the women in the test group met the proper distribution ranges for macronutrients, including proteins, fat, and carbohydrates, they fell short of the recommended intake levels of micronutrients, including vitamins D, B9, total fiber, and sodium [21]. The authors found that women with PCOS and those without the condition demonstrated similar dietary and physical activity behaviors, although those with the condition had a higher BMI. The findings supported the current evidence-based guidelines on the prevention and management of PCOS, which entails adopting and maintaining a nationally recommended healthy lifestyle, including diet and physical activities, for women with PCOS. Moreover, Lin et al. found that although people with PCOS met the dietary requirements for macronutrients, they scored decimally regarding fiber consumption and demonstrated significantly excessive sodium intake [21]. Despite the failure to find significant differences between the test and control groups regarding dietary and physical activity behaviors, the authors emphasize the need to promote lifestyle changes, mainly geared toward weight loss and maintenance, as an integral approach to the management of PCOS and its associated metabolic comorbidities [21]. A healthy lifestyle as an effective approach to PCOS management has been described in other studies, with or without clear findings on the association between diet and disease incidence [3,9,10,13].

In yet another study, Shahdadian et al. sought to understand the association between dietary habits and the incidence of PCOS [22]. They used a case-controlled design with 225 women recently diagnosed with PCOS against the control of 345 healthy individuals. Using a 168-item questionnaire to determine food frequency, the authors identified three dietary patterns, including western, plant-based, and mixed categories [22]. The Western category comprised organ meats, soft drinks, high-fat dairy, fast foods, salty snacks, desserts, sweets, sugar, mayonnaise, and other processed foods [22]. On the other hand, plant-based diets included legumes, fruits, yogurt, canned fruits, vegetables, nuts, fruit juices, garlic, potatoes, and condiments. Finally, the mixed-diet patterns entailed consuming foods drawn from both the Western and plant-based categories [22].

The influence of cultural and regional dietary habits on the prevalence of PCOS has also been established. Abruzzese et al. discovered that PCOS among women in most Latin American populations is shaped by the dietary habits inherited through their ancestry, further compounded by their socioeconomic status [23]. Given that a majority of Latin American countries fall within the low- to lower-middle economic bracket, the lifestyle and dietary choices of their inhabitants are significantly impacted by their socioeconomic circumstances, consequently contributing to the prevalence of PCOS in this region [23]. The authors also highlight the distinction between urban and rural dwellers, who lead contrasting lifestyles and possess diverse cultural backgrounds. As a result, these factors play a role in how diet affects PCOS.

Shishehgar et al. argue that dietary patterns are strongly influenced by the predominant culture of a region [24]. Given the links established between specific food types and conditions, such as T2DM and cardiovascular disease, investigating the impact of diet on PCOS holds significance. The authors also attribute the apparent discrepancies in certain study findings, particularly those that seemingly indicate no disparity in BMI and waist circumference between test and control groups, to the differing food habits prevalent in various cultures [24]. In another study elucidating the role of regional lifestyles in PCOS, Hajivandi et al. revealed that adolescent girls with PCOS in Iran exhibited a high intake of fatty and salty foods, sugar-rich items, and unhealthy snacks, coupled with low consumption of fiber, dairy products, fish, seafood, and legumes [25]. The authors noted that participants frequently skipped meals while consuming large portions per sitting. Recognizing the influence of regional and cultural dietary practices is pivotal in devising appropriate interventions tailored to different demographic groups.

Shahdadian et al. demonstrated that the Western dietary pattern was associated with an increased risk of PCOS. The Western category of foods includes a lot of simple sugar and trans and saturated fatty acids, thus increasing the incidence of obesity and insulin resistance [22]. Western foods were also associated with a high concentration of animal protein, which, unlike plant proteins, increases the concentration of insulin-like growth factor (IGF-I) in the blood, which is associated with a high risk of PCOS through the increased production of ovarian theca-interstitial cells [22].

On the other hand, the study indicated that a plant-based diet could have a protective effect against PCOS [22]. However, after adjusting for confounders such as fiber and antioxidant vitamin intake and physical activity, the supposed protective effects were no longer evident [22]. In other words, there was a non-linear association between a plant-based dietary pattern and the risk of PCOS, which the authors associated with the use of pesticides in agriculture. Shahadian et al. further found that moderate adherence to a mixed dietary pattern may have a protective effect on the risk of PCOS [22]. However, participants who exhibited high-level intake of this category of foods did not have an increased advantage regarding the prevention of PCOS. The researchers considered this observation to be due to the fact that even in the mixed dietary group, healthy and unhealthy foods may overlap, thus limiting the ability to conclusively declare a protective impact for those adhering to this pattern [22]. Therefore, they concluded that both western and plant-based dietary patterns are linked to a high risk of PCOS, while a moderate mixed dietary pattern may lead to a lower risk of the condition, but the findings can only be confirmed with more studies following a longitudinal design [22].

Despite the majority of studies linking dietary habits to PCOS and finding that most women with the condition are obese, researchers have also discovered a phenomenon where PCOS women are either normal-weight or even underweight [26]. As a result, the concept of "lean PCOS" has been adopted to describe such cases. Deswal et al. found a largely similar prevalence among both obese and lean groups. However, their study also revealed a higher rate among obese women who also had insulin resistance [1]. Because the disease has traditionally been considered to primarily affect obese women, the proportion of individuals with lean PCOS might be neglected both in diagnosis and management.

Toosy et al. describe the features of lean PCOS and propose diagnostic and treatment approaches based on their literature review findings [26]. They found that approximately 80% of PCOS cases are obese or have a BMI above the normal range, suggesting that the prevalence of lean PCOS is about 20%. They note that while this proportion is relatively smaller compared to the obese group, it is still significant since affected women may present with symptoms such as irregular menses and acne [26]. The authors point out that the clinical presentation of lean PCOS is similar to the overweight phenotypes, including the presence of dysmenorrhea, endometrial hyperplasia, and hirsutism for PCOS women affected by infertility. Furthermore, abnormal hormonal profiles, including an altered LH to follicle-stimulating hormone (FSH) ratio, serum levels of progesterone and LH, and elevated testosterone, were observed in lean cases similar to overweight instances [26]. Additionally, evidence shows that women with lean PCOS are also insulin-resistant, leading to hyperinsulinemia. Understanding these features is crucial to ensuring that the lean PCOS phenotype is not overlooked.

The presence of hyperinsulinemia in lean PCOS patients was also noted by Barrea et al., who indicate that beta (β)-cell dysfunction could be an outcome independent of a person’s weight [27]. The authors note that while lean PCOS women exhibit lower instances of insulin resistance and serum insulin levels compared to their obese counterparts, these parameters are still significantly elevated in relation to healthy individuals. They consider the possibility of hypersecretion of insulin as a major cause of changes in both obese and lean cases, with the most likely factor being adiponectin down-regulation, potentially resulting in insulin resistance. Altered lipid profiles are another observation in both lean and obese PCOS women, although with slight differences. According to Barrea et al., women with lean PCOS present lower high-density lipoprotein (HDL) and HDL2 levels compared to healthy individuals with normal ovarian functionality [27]. Similarly, lean PCOS cases are as likely to be affected by oxidative stress and closely related low-grade inflammation as their obese PCOS counterparts. Therefore, despite some slight but undeniable differences in symptom severity, the features of lean PCOS are as significant as in overweight phenotypes.

Toosy et al. affirm that the severity of symptoms may differ between the two groups [26]. The authors argue that obese PCOS women experience more severe metabolic and hormonal derangements compared to lean PCOS patients. They found that the rate of hyperandrogenism was 74.2% for obese cases compared to 50.6% for lean women, while menstrual dysfunction was present in 79.3% of overweight women compared to 44% of lean PCOS women [26]. Similarly, there was a higher prevalence of T2DM and impaired glucose tolerance among obese women compared to their lean counterparts. These findings indicate a significant difference in symptom severity between the two phenotypes. Toosy et al. note that the observations indicate that obese PCOS women may be at a higher risk of developing clinical PCOS at a younger age compared to the lean cohort [26]. They may also require a more rigorous treatment approach.

## Conclusions

This review concludes by establishing an unequivocal link between dietary habits and the onset of PCOS. The majority of the reviewed studies consistently demonstrate that women with PCOS share similar dietary behaviors, characterized by a heightened consumption of unhealthy food items, including carbohydrates, animal proteins, fats, and processed foods. This consensus persists despite certain studies presenting contradictory findings that suggest an unclear and inconsistent association between nutrition and PCOS. Additionally, there appears to be a consensus that lifestyle modification is crucial to preventing and managing PCOS. In particular, weight loss and maintenance are shown to be vital to the prevention of the condition. This review has shown an increased risk of PCOS with Western dietary patterns and high-fat, low-fiber diets. Other studies evaluated in this paper have indicated that higher GI and GL foods increase the risk of PCOS. The AHEI diet decreases the risk of PCOS hyperandrogenism and oligo anovulation phenotype. There appears to be a relationship between diet and different PCOS phenotypes. The prevalence of PCOS is also shown to be affected by micronutrients, including vitamin D and B9. It has also been demonstrated that a genetic-environmental interaction influences the occurrence of PCOS. When genetically predisposed women are exposed to detrimental environmental factors, such as an unhealthy diet and inactivity, they are more likely to develop the condition. This study also unveiled a phenomenon referred to as "lean PCOS," where 20% of global PCOS cases display clinical manifestations of the condition despite having either a normal weight or being underweight. All in all, the existence of studies with conflicting findings underscores the necessity for additional research involving substantial cohorts.

## References

[1] Deswal R, Narwal V, Dang A, Pundir CS (2020). The prevalence of polycystic ovary syndrome: a brief systematic review. J Hum Reprod Sci.

[2] Sedighi S, Amir Ali Akbari S, Afrakhteh M, Esteki T, Alavi Majd H, Mahmoodi Z (2014). Comparison of lifestyle in women with polycystic ovary syndrome and healthy women. Glob J Health Sci.

[3] Mani H, Levy MJ, Davies MJ (2013). Diabetes and cardiovascular events in women with polycystic ovary syndrome: a 20-year retrospective cohort study. Clin Endocrinol (Oxf).

[4] Cooney LG, Lee I, Sammel MD, Dokras A (2017). High prevalence of moderate and severe depressive and anxiety symptoms in polycystic ovary syndrome: a systematic review and meta-analysis. Hum Reprod.

[5] Azziz R, Carmina E, Dewailly D (2006). Positions statement: criteria for defining polycystic ovary syndrome as a predominantly hyperandrogenic syndrome: an Androgen Excess Society guideline. J Clin Endocrinol Metab.

[6] The Lancet Regional Health-Europe (2022). Polycystic ovary syndrome: what more can be done for patients?. Lancet Reg Health Eur.

[7] (2022). PCOS (polycystic ovary syndrome) and diabetes. https://www.cdc.gov/diabetes/basics/pcos.html.

[8] Liu J, Wu Q, Hao Y, Jiao M, Wang X, Jiang S, Han L (2021). Measuring the global disease burden of polycystic ovary syndrome in 194 countries: Global Burden of Disease Study 2017. Hum Reprod.

[9] Legro RS, Driscoll D, Strauss JF 3rd, Fox J, Dunaif A (1998). Evidence for a genetic basis for hyperandrogenemia in polycystic ovary syndrome. Proc Natl Acad Sci U S A.

[10] Altieri P, Cavazza C, Pasqui F, Morselli AM, Gambineri A, Pasquali R (2013). Dietary habits and their relationship with hormones and metabolism in overweight and obese women with polycystic ovary syndrome. Clin Endocrinol (Oxf).

[11] Chavarro JE, Rich-Edwards JW, Rosner BA, Willett WC (2007). Diet and lifestyle in the prevention of ovulatory disorder infertility. Obstet Gynecol.

[12] Chavarro JE, Rich-Edwards JW, Rosner BA, Willett WC (2009). A prospective study of dietary carbohydrate quantity and quality in relation to risk of ovulatory infertility. Eur J Clin Nutr.

[13] Xenou M, Gourounti K (2021). Dietary patterns and polycystic ovary syndrome: a systematic review. Maedica (Bucur).

[14] Lin AW, Lujan ME (2014). Comparison of dietary intake and physical activity between women with and without polycystic ovary syndrome: a review. Adv Nutr.

[15] Scarfò G, Daniele S, Fusi J (2022). Metabolic and molecular mechanisms of diet and physical exercise in the management of polycystic ovarian syndrome. Biomedicines.

[16] Kshetrimayum C, Sharma A, Mishra VV, Kumar S (2019). Polycystic ovarian syndrome: environmental/occupational, lifestyle factors; an overview. J Turk Ger Gynecol Assoc.

[17] Parker J, O'Brien C, Hawrelak J, Gersh FL (2022). Polycystic ovary syndrome: an evolutionary adaptation to lifestyle and the environment. Int J Environ Res Public Health.

[18] Cutillas-Tolín A, Arense-Gonzalo JJ, Mendiola J (2021). Are dietary indices associated with polycystic ovary syndrome and its phenotypes? A preliminary study. Nutrients.

[19] Eslamian G, Baghestani AR, Eghtesad S, Hekmatdoost A (2017). Dietary carbohydrate composition is associated with polycystic ovary syndrome: a case-control study. J Hum Nutr Diet.

[20] Eslamian G, Hekmatdoost A (2019). Nutrient patterns and risk of polycystic ovary syndrome. J Reprod Infertil.

[21] Lin AW, Kazemi M, Jarrett BY, Vanden Brink H, Hoeger KM, Spandorfer SD, Lujan ME (2019). Dietary and physical activity behaviors in women with polycystic ovary syndrome per the new international evidence-based guideline. Nutrients.

[22] Shahdadian F, Ghiasvand R, Abbasi B, Feizi A, Saneei P, Shahshahan Z (2019). Association between major dietary patterns and polycystic ovary syndrome: evidence from a case-control study. Appl Physiol Nutr Metab.

[23] Abruzzese GA, Velazquez ME, Cerrone GE, Motta AB (2023). Polycystic ovary syndrome in Latin American populations: what is known and what remains unresolved. J Steroid Biochem Mol Biol.

[24] Shishehgar F, Ramezani Tehrani F, Mirmiran P, Hajian S, Baghestani AR, Moslehi N (2016). Comparison of dietary intake between polycystic ovary syndrome women and controls. Glob J Health Sci.

[25] Hajivandi L, Noroozi M, Mostafavi F, Ekramzadeh M (2020). Food habits in overweight and obese adolescent girls with Polycystic ovary syndrome (PCOS): a qualitative study in Iran. BMC Pediatr.

[26] Toosy S, Sodi R, Pappachan JM (2018). Lean polycystic ovary syndrome (PCOS): an evidence-based practical approach. J Diabetes Metab Disord.

[27] Barrea L, Frias-Toral E, Verde L (2021). PCOS and nutritional approaches: differences between lean and obese phenotype. Metabol Open.

